# Unterstützungsbedarfe pflegeempfangender Menschen: Erhebung allgemeiner und spezieller Informations‑, Hilfe- und Unterstützungsbedarfe pflegeempfangender Menschen und ihrer Angehörigen

**DOI:** 10.1007/s00391-022-02068-w

**Published:** 2022-05-04

**Authors:** Sandra Strube-Lahmann, Franziska Müller, Daniela Liersch-Mazan, Michele Haink, Ursula Müller-Werdan, Nils A. Lahmann

**Affiliations:** 1 Forschungsgruppe Geriatrie, AG-Pflegeforschung, Medizinische Klinik für Geriatrie und Altersmedizin der Charité – Universitätsmedizin Berlin, Reinickendorfer Straße 61, 13347 Berlin, Deutschland; 2 Fakultät Gesundheitswissenschaften, Medical School Berlin , Berlin, Deutschland; 3 Geschäftsbereich hochschulische Aus- und Weiterbildung, Akademie der Gesundheit Berlin/Brandenburg e. V., Schwanebecker Chaussee 4 E–H, 13125 Berlin, Deutschland

**Keywords:** Pflegestützpunkt, Pflegebedürftigkeit, Beratungsbedarf, Angehörige, Pflegeberatung, Care support point, Need for care, Need for advice, Relatives, Care consulting

## Abstract

**Hintergrund:**

Pflegeempfangende und deren Angehörige haben in Deutschland seit 2009 einen gesetzlichen Anspruch auf eine Pflegeberatung. Um Pflegeberatende angemessen auf aktuelle Beratungsbedarfe vorzubereiten, sind dafür zunächst die bestehenden Anliegen zu erfassen. Ziel der Studie war es, die unterschiedlichen Bedarfe von Pflegeempfangenden und deren Angehörigen zu ermitteln.

**Methodik:**

Es erfolgte eine Online-Befragung von Pflegeberatenden mittels standardisiertem Fragebogen. Die Ausprägung des allgemeinen Beratungsbedarfs von Pflegeempfangenden und Angehörigen wurde mittels 10-stufiger Skala (1 [gering] bis 10 [hoch]) erhoben. Über eine 5‑stufige Likert-Skala wurde die Höhe vorliegender Beratungsbedarfe zu 16 Themenschwerpunkten bewertet. Mittels „classification and regression trees“ (CRT) und „random forest“ wurde der Zusammenhang zwischen den einzelnen Themenschwerpunkten und dem allgemeinen Beratungsbedarf analysiert.

**Ergebnisse:**

Der allgemeine Informationsbedarf von Pflegeempfangenden bzw. Angehörigen wurde von den teilnehmenden Pflegeberatern (*n* = 276) mit MW 7,8 bzw. MW 9,2 eingeschätzt. Bei den Pflegeempfangenden bestand laut CRT der stärkste Zusammenhang zwischen allgemeinem Informationsbedarf und „Wohnraumberatung“, bei den Angehörigen zwischen allgemeinem Informationsbedarf und „Sozialrechtliche[n] Aspekte[n], Ansprüche[n], Leistungen“.

**Diskussion:**

Der allgemeine Informationsbedarf von Pflegeempfangenden und Angehörigen wird als sehr hoch eingeschätzt. Dennoch lassen sich Unterschiede feststellen. Daher erscheint es notwendig, Pflegeberatungen nach den gruppenspezifischen Beratungsbedarfen auszurichten.

**Zusatzmaterial online:**

Zusätzliche Informationen sind in der Online-Version dieses Artikels (10.1007/s00391-022-02068-w) enthalten.

## Abstract (en)

**Background:**

In 2009, statutory regulations on information and counselling regarding nursing care needs, performed by so-called care advisors have been implemented for persons in need of long-term care and their relatives. In order to adequately prepare these care advisors, contemporary needs and requirements must be determined. The aim of the study was to determine the different needs of persons in need of long-term care and their relatives.

**Method:**

Care advisors were interviewed via an online survey tool using a standardized questionnaire. A 5-point Likert scale was used to determine the needs regarding information and advice on 16 specific topics. In general, overall needs regarding information and advice of care recipients and relatives were recorded using a 10-point scale (1 low and 10 high). Using classification and regression trees (CRT) and random forest, the correlation between the individual main topics and the general need for advice was analyzed.

**Results:**

The participating care advisors (*n* = 276) rated the general demand for information of people in need of care and their relatives with a mean of 7.8 and 9.2, respectively. For those in need of care, the strongest association of general information needs was the topic of housing advice For the relatives, the topic social law aspects and benefits was the most relevant association.

**Conclusion:**

The general demand for information was rated very high. Since differences became obvious between those in need of care and their relatives, it is necessary to adjust care advice for these two groups.

Eine Pflegeberatung hat das Ziel, pflegeempfangende Menschen und deren Angehörige zu unterstützen. Sie soll allen Beteiligten einen gleichberechtigten Zugang zu bedarfsgerechten und individuellen Hilfs‑, Unterstützungs- und Versorgungsleistungen ermöglichen. Sowohl Quantität als auch Qualität der Beratungsangebote sind in Deutschland derzeit eher heterogen. Vor diesem Hintergrund wurde eine deutschlandweite Untersuchung durchgeführt, deren Ergebnisse einen Beitrag leisten sollen, die Beratungsqualität zu stärken. Dafür sind zunächst die entsprechenden Beratungsbedarfe zu ermitteln.

Die Pflegeberatung ist elementarer Bestandteil in der Gesundheitsversorgung pflegeempfangender Menschen sowie von deren Angehörigen. Sie soll zur Erfassung und zur Sicherung des individuellen Pflege- und Versorgungsbedarfes beitragen [11]. Sie hat zum Ziel, Menschen mit Pflege‑, Versorgungs- und Unterstützungsbedarf so mit Informationen zu versorgen, dass Betroffene selbstbestimmt über verschiedene Angebote entscheiden können, und ist im Sozialgesetzbuch (SGB) XI § 7a klar definiert [3]. Dabei beschränkt sich die Beratung nicht nur auf die pflegeempfangende Person selbst, sondern auf deren Wunsch hin auch auf Angehörige und weitere Personen [2]. Hierbei sieht das SGB XI verschiedene Möglichkeiten der Schulung, Beratung und Aufklärung, insbesondere mit dem Fokus der Versorgung in der eigenen Häuslichkeit, vor [1, 3]. So haben Pflegeempfangende und deren Angehörige in Deutschland nach § 7a SGB XI einen gesetzlichen Anspruch auf eine individuelle, unabhängige und umfassende Pflegeberatung, welche nicht zuletzt auch präventive Orientierung geben soll. Zur gezielten Stärkung der häuslichen Versorgung und Sicherung der entsprechenden Versorgungsqualität werden darüber hinaus verpflichtende, regelmäßige Beratungsbesuche nach § 37 Abs. 3 SGB XI für Pflegegeldempfangende durchgeführt. Vor dem Hintergrund der kontinuierlichen Zunahme pflegeempfangender Menschen (derzeit ca. 4 Mio.), welche überwiegend durch pflegende Angehörige in der eigenen Häuslichkeit (ca. 80 %) versorgt werden [8], gilt die Pflegeberatung als unverzichtbares Instrument, Betroffenen und deren Angehörigen einen gleichberechtigen und verbesserten Zugang zu Sozialleistungen, Präventionsmaßnahmen und bedarfsgerechten Unterstützungsleistungen zu ermöglichen [11]. Hierbei ist diese in den vergangenen Jahren sukzessive ausgebaut und optimiert worden. Mithilfe der benannten Beratungsmöglichkeiten und der Bereitstellung eines nahezu deutschlandweiten Netzes von Pflegestützpunkten sollen der Zugang zu entsprechenden Hilfeleistungen sowie die Inanspruchnahme bestehender Unterstützungsangebote erleichtert werden [10]. Beratungseinsätze, die durch frei wählbare, zugelassene Pflegedienste oder von einer durch die Pflegekasse anerkannten pflegefachlichen Beratungsstelle erfolgen, sollen hierbei insbesondere die Qualität der häuslichen Pflege gewährleisten bzw. Fragen der Betroffenen im Hinblick auf die Pflegesituation klären [12]. Für die komplexe Beratungstätigkeit sind Pflegeberatende, entsprechend den geltenden Empfehlungen, umfassend zu qualifizieren [11]. Denn insbesondere sie sind häufig die erste Anlaufstelle für Pflegempfangende und deren Angehörige. Bei allen Fragen rund um das Thema Pflege sollen sie unterstützen, über Ansprüche informieren und über passende Unterstützungsangebote aufklären können [16]. In Bezug auf die Qualität der Beratungen ist die Studienlage inhomogen. So wird aufgrund von Untersuchungen empfohlen, Beratungsangebote verstärkt an Qualitätsstandards auszurichten und hierbei die Beratungsanlässe und -bedarfe aus der Perspektive der Nutzenden aufzugreifen [5, 9, 10]. Andere Studien zeigten, dass sich die meisten Ratsuchenden zum Thema Hilfs- und Unterstützungsangebote im Pflegebereich weniger gut informiert fühlten, jedoch der allgemeine Informations- und Beratungsbedarf von Betroffenen als sehr hoch eingeschätzt wurde. Darüber hinaus wird auf eine heterogene Kompetenz- und Qualitätsstruktur der Pflegeberatenden verwiesen [17]. Eine Studie des Institut für Gesundheits- und Sozialforschung (IGES) zeichnet hingegen ein positives Bild sowohl im Hinblick auf Beratungskompetenz als auch in Bezug auf Beratungsinhalte. Fast alle Beratungssuchenden wussten nach einem Beratungsgespräch, was als Nächstes zu erledigen wäre [14]. Ratsuchende gaben nach Beratungsgesprächen in Pflegekassen mit deutlicher Mehrheit an, dass sich ihr Wissen über Leistungsmöglichkeiten durch die Beratung deutlich verbessert habe [6]. Die mit Blick auf Qualität und Quantität der Beratungsangebote sehr heterogene Studienlage ist möglicherweise auf die sehr unterschiedliche Ausgestaltung einzelner Kompetenzbereiche auf Länderebene zurückzuführen. Um die Qualität der Beratung auf einem hohen Niveau zu standardisieren, müssen die entsprechenden Bedarfe zunächst umfassend ermittelt werden [6]. Folglich war das *Ziel *dieser Studie, den allgemeinen und speziellen Informations‑, Hilfe- und Unterstützungsbedarf von Pflegeempfangenden und deren Angehörigen/Betreuenden zu ermitteln.

## Methodik

### Stichprobe und Datenerhebung

Im Frühjahr 2021 wurde mittels SoSci Servey-Erhebungstool eine Querschnitterhebung Pflegeberatender durchgeführt. Dazu wurden die frei im Internet verfügbaren Kontaktdaten entsprechender Pflegeberatungsstellen genutzt. Zusätzlich wurde der Umfragelink durch bestehende Netzwerke verbreitet. Dies erfolgte per Mail bzw. per Social Media und durch telefonische Kontaktaufnahme zu Pflegeberatenden in Pflegestützpunkten, Pflegekassen und weiteren Pflegeberatungseinrichtungen. Einschlusskriterium war eine derzeitige Tätigkeit als Pflegeberater bzw. Pflegeberaterin nach § 7a und § 37 SGB XI [12]. Die Teilnehmenden erhielten Informationen zur Studie und einen Link zur Online-Befragung. Der Fragebogen wurde im Vorfeld mittels Pretest auf Plausibilität, Verständlichkeit und Lesbarkeit bei insgesamt *n* = 5 Pflegenden/Pflegeberatenden geprüft. Auf deren Grundlage wurden unklare Formulierungen korrigiert.

### Erhobene Daten

Es wurden soziodemografische Daten der Pflegeberatenden, wie Alter, Geschlecht, Berufsausbildung und Qualifikation, höchster erreichter Bildungsabschluss, Berufserfahrung in der praktischen Pflege und Berufserfahrung in der Pflegeberatung in Jahren erhoben. Die Pflegeberatenden sollten ihren subjektiven Gesamteindruck zu den 16 Themenschwerpunkten (Tab. 1) der Beratungssuchenden einschätzen. Die Erfassung des Beratungsbedarfes erfolgte anhand einer 5‑stufigen Likert-Skala von sehr gering (1) bis sehr hoch (5). Die jeweiligen Themenschwerpunkte wurden mit entsprechenden Beispielen im Fragebogen konkretisiert, wie z. B. Thema „Pflegebedürftigkeit/pflegerische Versorgung“ (z. B. Antrag Pflegegrad/Erhöhung Pflegegrad, Überleitungspflege, individueller Versorgungsplan). Diese wurden auf Basis einer umfassenden Literaturrecherche ermittelt. Schließlich sollten die Pflegeberatenden auf einer Skala von 1 (sehr gering) bis 10 (sehr hoch) den allgemeinen Informationsbedarf zum einen von Pflegeempfangenden und zum anderen von deren Angehörigen/Betreuenden im Rahmen der Pflegeberatung einschätzen.

**Table 1:** 

Thema		Sehr gering (1)	Gering (2)	Mittel (3)	Hoch (4)	Sehr hoch (5)	Gesamt	Mittelwert	Standardabweichung	Median
		%	%	%	%	%	*n*	MW	SD	MD
1	Gesundheitliche Situation	1,9	4,1	20,1	46,3	27,6	268	3,9	0,9	4,0
2	Pflegebedürftigkeit/pflegerische Versorgung	1,5	0,4	4,1	28,5	65,5	267	4,6	0,7	5,0
3	Mobilität	1,5	6,0	34,8	44,6	13,1	267	3,6	0,8	4,0
4	Ambulante Leistungen	1,5	0,4	4,5	34,5	59,2	267	4,5	0,7	5,0
5	Stationäre Leistungen	4,1	10,8	39,6	26,1	19,4	268	3,5	1,1	3,0
6	Heil- und Hilfsmittel	1,9	6,7	28,4	44,0	19,0	268	3,7	0,9	4,0
7	Medizinische Leistungen	6,3	19,0	44,0	22,4	8,2	268	3,1	1,0	3,0
8	Psychosoziale Situation	1,9	6,7	17,5	35,8	38,1	268	4,0	1,0	4,0
9	Selbstbestimmung und Persönlichkeitsrechte	15,7	34,3	25,7	18,3	6,0	268	2,7	1,1	2,5
10	Prävention, gesellschaftliche und soziale Teilhabe	7,4	33,5	32,7	19,0	7,4	269	2,9	1,0	3,0
11	Rehabilitation	12,7	32,8	33,2	15,3	6,0	268	2,7	1,1	3,0
12	Finanzielle und hauswirtschaftliche Situation	1,5	1,5	10,4	40,7	45,9	268	4,3	0,8	4,0
13	Wohnraumberatung	1,5	3,7	28,7	47,8	18,3	268	3,8	0,8	4,0
14	Sozialrechtliche Aspekte, Ansprüche, Leistungen	1,1	3,4	19,4	37,7	38,4	268	4,1	0,9	4,0
15	Kulturelle und religiöse Aspekte	25,7	38,8	25,7	6,3	3,4	268	2,2	1,0	2,0
16	Aktivitäten des täglichen Lebens	7,8	23,5	35,4	25,0	8,2	268	3,0	1,1	3,0

### Datenanalyse

Die Beschreibung der Ergebnisse erfolgte zunächst deskriptiv in absoluten und relativen Häufigkeiten. Für metrische Daten wurden Mittelwerte (MW) und Standardabweichungen (SD) berechnet. Zur Identifizierung der Beziehungen der 16 Themenschwerpunkte zum allgemeinen Informationsbedarf der Pflegeempfangenden und deren Angehöriger wurden multivariate Analysen durchgeführt. Hierbei wurde mittels „Classification-and-regression-trees“(CRT)-Verfahren und „Random-forest“-Statistiken der Zusammenhang der unabhängigen Variablen (16 Themenschwerpunkte) in Hinblick auf die jeweils abhängigen Variablen (allgemeinen Informationsbedarfe Pflegeempfangende und Angehörige/Betreuende) ermittelt. Für die Berechnungen wurden die Statistikprogramme SPSS (IBM Corp. Released 2015. IBM SPSS Statistics for Windows, Version 23.0. Armonk, NY: IBM Corp) der International Business Machines Corporation 23 und „R“ (entwickelt von: R Core Team) genutzt.

## Ergebnisse

An der Untersuchung haben 274 Pflegeberatende teilgenommen. Der überwiegende Anteil (81 %) der Pflegeberatenden war weiblichen Geschlechts. Angaben zu Alter und Bildungsabschluss lagen von 267 Personen vor. Die meisten Befragten waren zwischen 50 und 59 (30,3 %) bzw. 40 und 49 (21,9 %) Jahre alt. Über eine 3‑jährige Berufsausbildung verfügten 32,5 %; einen Bachelorabschluss hatten 28,5 %, einen Masterabschluss 11,3 %. Von 268 Pflegeberatenden waren 118 Personen (43,1 %) länger als 10 Jahre und 34 (12,4 %) Personen 5 bis 10 Jahre in der praktischen Pflege tätig (weitere Informationen zur Stichprobe: Zusatzmaterial online, Teil 1).

In Tab. 1 wird dargestellt, wie hoch Pflegeberatende den an sie herangetragenen Beratungsbedarf zu den 16 Themenschwerpunkten einschätzen. Hoch bis sehr hoch schätzten Pflegeberatende den an sie herangetragenen Beratungsbedarf am häufigsten bei den Themenbereichen „Pflegebedürftigkeit/pflegerische Versorgung“ (28,5 %; 65,5 %) und „Ambulante Leistungen“ (34,5 %; 59,2 %). Der geringste Beratungsbedarf (gering bzw. sehr gering) bestand aus Sicht der Befragten bei den Themen „Kulturelle und religiöse Aspekte“ (38,8 %; 25,7 %) und „Rehabilitation“ (32,8 %; 12,7 %).

**Figure 1:**
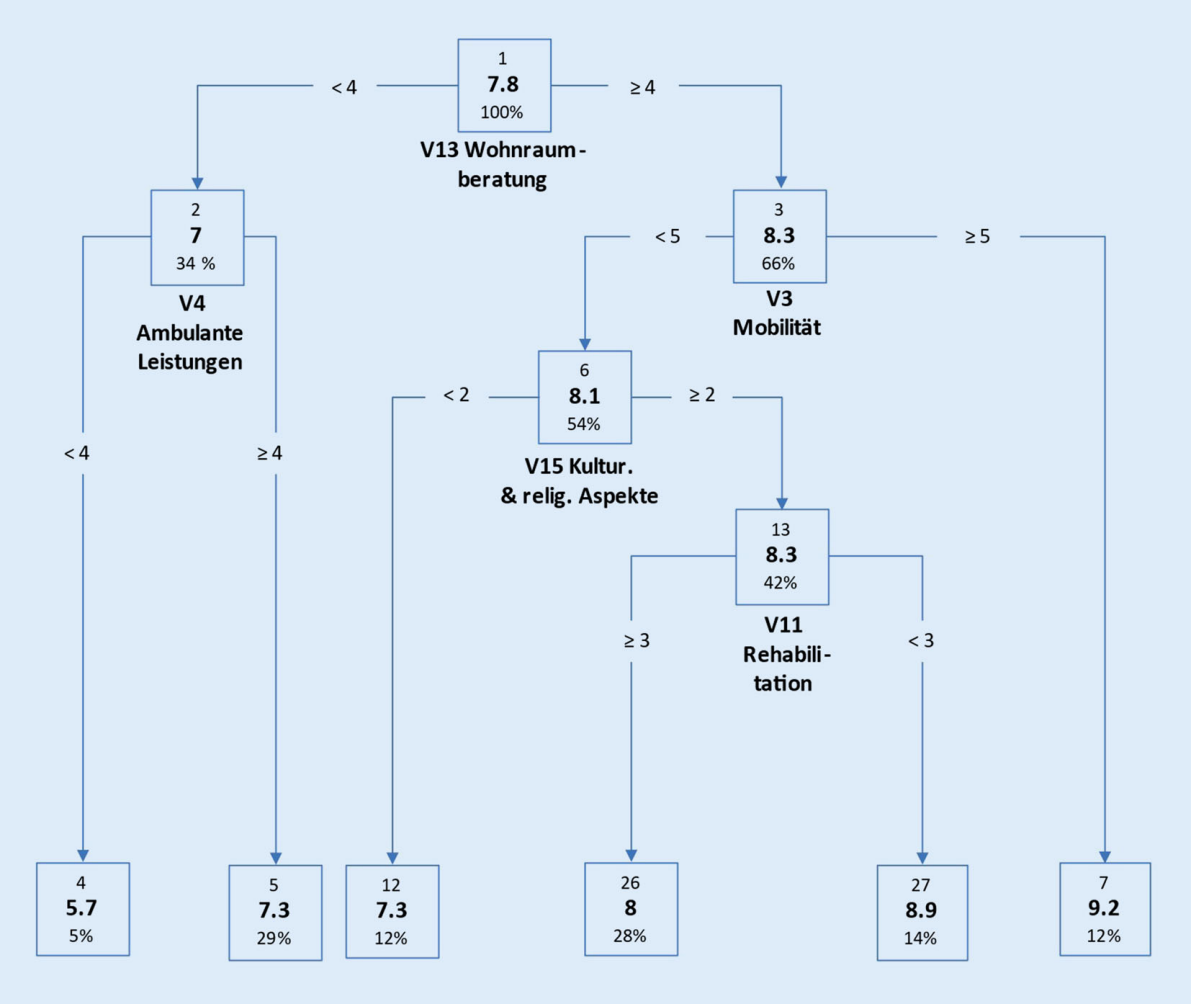

*Der allgemeine Informationsbedarf* von Pflegeempfangenden bzw. Angehörigen/Betreuenden wurde durch die Pflegeberatenden mit MW 7,8 bzw. MW 9,2 eingeschätzt. Die Abb. 1 veranschaulicht mittels CRT den Zusammenhang der 10-stufigen Zielgröße „Allgemeiner Informationsbedarf von Pflegeempfangenden“ (AIPE) mit den 16 Themenbereichen. Im Gegensatz zu anderen Techniken werden in den baumbasierten Modellen die optimalen Aufteilungen der inhomogenen Gesamtstichprobe auf Basis der 16 Themenbereiche sukzessive in „homogenen“ Teilmengen dargestellt. Es zeigte sich, dass 5 der 16 Themenbereiche (V13 = „Wohnraumberatung“, V3 = „Mobilität“, V4 = „Ambulante Leistungen“, V15 = „Kulturelle und religiöse Aspekte“(Kultur.& relig. Aspekte), V11 = „Rehabilitation“) eine bedeutende Rolle für die Bildung der endgültigen 6 Knotenpunkte (4, 5, 12, 26, 27, 7) haben.

Ausgehend von der gesamten Stichprobe (Knoten Nr. 1, MW = 7,8) erwies sich der Themenbereich „Wohnraumberatung“ für den ersten Split als stärkster Prädiktor (detaillierte Ergebnisbeschreibung: Zusatzmaterial online, Teil 2).

**Figure 2:**
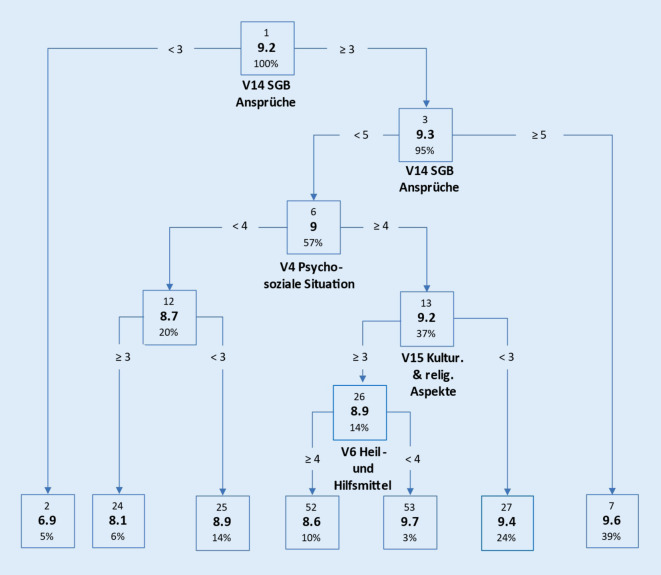

Abb. 2 veranschaulicht mittels CRT den Zusammenhang der 10-stufigen Zielgröße „Allgemeiner Informationsbedarf der Angehörigen/Betreuenden“ (AIAB) mit den 16 Themenbereichen. Von diesen spielen 5 Bereiche (V14 = „Sozialrechtliche Aspekte, Ansprüche, Leistungen (SGB XI, Betreuungsrecht (SGB Ansprüche))“, V8 = „Psychosoziale Situation“, V9 = „Selbstbestimmung und Persönlichkeitsrechte“, V15 = „Kulturelle und religiöse Aspekte“, V6 = „Heil- und Hilfsmittel“) für die Bildung der endgültigen 7 Knotenpunkte (2, 24, 25, 52, 53, 27, 7) eine bedeutende Rolle. Für den ersten Split, ausgehend von der Gesamtstichprobe (Knoten 1, MW 9,2), erwies sich der Themenbereich „Sozialrechtliche Aspekte, Ansprüche, Leistungen (SGB XI, Betreuungsrecht)“ als stärkster Trenner der Stichprobe (detaillierte Beschreibung der Analyse: Zusatzmaterial online, Teil 3).

**Figure 3:**
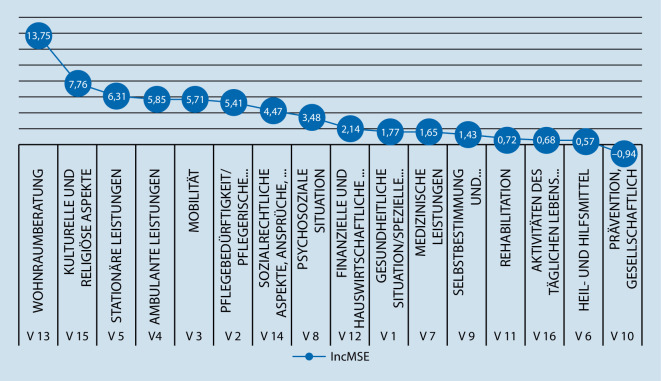

**Figure 4:**
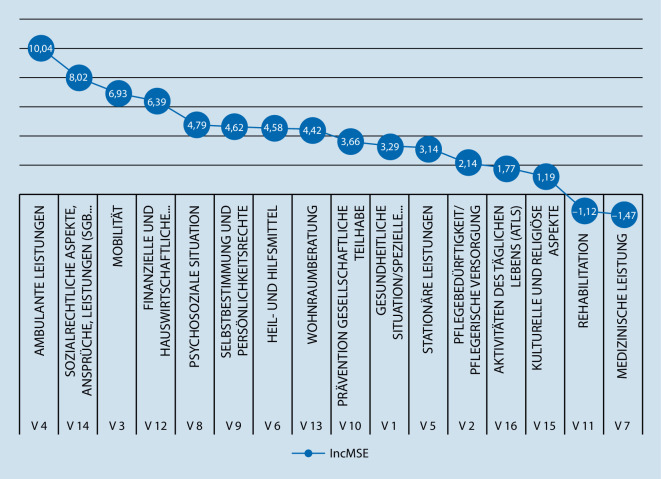

Während die Baumdarstellungen nur die lokal signifikanten trennenden Merkmale zeigen, wird mit der R‑routine random forest die relative Bedeutung aller Themenschwerpunkte dargestellt. Im Ergebnis des Random forest zeigten sich für den AIPE (Abb. 3) die höchsten IncMSE bei den Themen „Wohnraumberatung“ (13,73), gefolgt von den Themen „Kulturelle und religiöse Aspekte“ (7,75), „Stationäre Leistungen“ (6,31), „Ambulante Leistungen“ (5,85) „Mobilität“ (5,71) sowie „Pflegebedürftigkeit/pflegerische Versorgung“ (5,41). Die geringsten IncMSE fielen bei den Themen „Prävention, gesellschaftliche und soziale Teilhabe“ (−0,94), „Heil- und Hilfsmittel“ (0,57), „Aktivitäten des täglichen Lebens (ATL)“ (0,68) sowie „Rehabilitation“ (0,72) auf. Beim AIAB (Abb. 4) zeigten sich der höchste IncMSE beim Thema „Ambulante Leistungen“ (10,04), gefolgt von „Sozialrechtliche Aspekte, Ansprüche, Leistungen (SGB XI, Betreuungsrecht)“ (8,02), „Mobilität“ (6,92) und „Wohnraumberatung“ (6,34). „Medizinische Leistungen“, „Rehabilitation“, „Kulturelle und religiöse Teilhabe“ sowie „Aktivitäten des täglichen Lebens (ATL)“ wiesen hingegen beim AIAB den geringsten IncMSE auf.

## Diskussion

Die teilnehmenden Pflegeberatenden schätzten den allgemeinen Informationsbedarf bei den Pflegeempfangenden als hoch, bei den Angehörigen sogar als sehr hoch ein. Diese Einschätzung könnte darauf zurückzuführen sein, dass Angehörige auch die besonders multimorbiden schwer und schwerstpflegebedürftigen Menschen versorgen, die selbst nicht mehr in der Lage sind, eine Pflegeberatung in Anspruch zu nehmen. Auch wenn Pflegeberatende insgesamt sehr häufig zu den Themenschwerpunkten „Sozialrechtliche Aspekte, Ansprüche/Leistungen“, „Finanzierung“, „Pflegebedürftigkeit/pflegerische Versorgung“ und, „Ambulante Leistungen“ um Auskunft gebeten wurden, zeigten die vorgenommenen Analysen durchaus Unterschiede im Beratungsbedarf zwischen den beiden Gruppen. Ziel der Analyse war es, die 16 Themenschwerpunkte in Zusammenhang mit der Gesamtbeurteilung des allgemeinen Informationsbedarfs von Pflegeempfangenden zum einen *und* den Angehörigen zum anderen zu setzen. Hier zeigt sich der Vorteil der verwendeten statistischen Modellierungen von Klassifikations- und Regressionsbäumen (CRT) sowie Random forest. Im Gegensatz zur Regressionsanalyse erfordert die CRT-Methode keine Normalverteilung der Datenresiduen. Darüber hinaus wäre die Anwendung von Regressionsmodellen wegen der hohen Korrelation der im Modell einbezogenen Variablen untereinander aufgrund der Multikollinearität problematisch.

Die durchgeführten Baumanalysen teilen die Stichprobe in typische Untergruppen ein. Diese Typisierung erlaubt auch ohne tiefgreifendes statistisches Verständnis eine sehr „plastische“ und nachvollziehbare Vorstellung dieser Gruppen. So wird deutlich, dass die beiden größten Untergruppen in Abb. 1 bei Pflegeempfangenden diejenigen sind, die eher weniger an Interesse an Wohnraumberatung haben, aber sehr hohes Interesse an ambulanten Leistungen. Dieses trifft fast auf ein Drittel der beschriebenen Population zu (Abb. 1, *Knoten 5*). Ähnliches gilt für die Untergruppe, die ein eher höheres Interesse an Wohnraumberatung und kulturellen und religiösen Aspekten aufweist, bei gleichzeitig geringerem Interesse an Mobilität und Rehabilitation (Abb. 1, *Knoten 26*). Auch diese Untergruppe umfasst beinahe ein Drittel der beschriebenen Population. Analog dazu sind die Ergebnisse aus Abb. 2 zu interpretieren, bei denen fast 40 % der beschriebenen Population ein sehr hohes Interesse gegenüber den Ansprüchen zum SGB haben (Abb. 2, *Knoten 7*). Während Pflegeempfangende nach Einschätzung der Pflegeberatenden eher die Themen „Wohnraumberatung“ sowie „Kulturelle und religiöse Aspekte“ adressierten, waren diese für Angehörige von nachrangiger Bedeutung. Vielmehr standen für sie die Themen „Ambulante Leistungen“ und „Sozialrechtliche Aspekte, Ansprüche, Leistungen (SGB XI, Betreuungsrecht)“ im Vordergrund. Hierbei ist anzunehmen, dass dies die priorisierte Rangfolge der relevanten Schwerpunktthemen der – die Pflegeberatung in Anspruch nehmenden – Pflegeempfangenden bzw. Angehörigen widerspiegelt. So müssen pflegende Angehörige die (eigene) finanzielle Situation klären, wenn sie aufgrund der Versorgung pflegeempfangender Angehöriger weniger oder nicht mehr ihrer eigentlichen Berufstätigkeit nachgehen können bzw. die finanzielle Belastung aufgrund der pflegerischen Versorgung in der Familie ansteigt. Pflegeempfangende hingegen müssen sich aufgrund der zu erwartenden Zunahme der eigenen Pflegebedürftigkeit mit der jeweils individuellen Wohnraumgestaltung auseinandersetzen, um Informationen zu bedarfsgerechten barrierefreien Wohnraumlösungen zu erhalten. Darüber hinaus treten für sie Beratungsbedarfe zu kulturellen und religiösen Aspekten in den Vordergrund. Inwieweit derzeit kulturelle und religiöse Aspekte im Rahmen von Beratungsbedarfen umfassend Berücksichtigung fanden, kann auf Basis dieser Studie nicht abschließend geklärt werden. Andere Untersuchungen zeigen jedoch, dass Beratungsangebote für Pflegeempfangende mit Migrationshintergrund generell verbessert werden sollten, da derzeit nur ca. ein Drittel der Beratungsstellen und in ungefähr der Hälfte der ländlichen und in jeder fünften städtischen Region keine speziellen Beratungsangebote für diese Zielgruppe angeboten werden [15]. Dies ist vor dem Hintergrund, dass der Anteil von Menschen mit Migrationshintergrund in der Bevölkerung zunimmt, besonders bedeutsam [4, 7]. Auf Grundlage dieser Analyse kann beim Aufbau einer Pflegeberatungsdatenbank zielgerichteter auf die Beratungsbedarfe einzelner Gruppen eingegangen werden, was einen positiven Effekt auf die Qualität der Pflegeberatung erwarten lässt. So könnten für Pflegeempfangende die Beratungsangebote zu Wohnraumberatung und kulturellen und religiösen Aspekten ausgebaut werden, für Angehörige hingegen mehr Informationen zu sozialrechtlichen Aspekten, Ansprüchen und Leistungen bereitgestellt werden. Auch mit Blick auf kurzfristig entstehende Bedarfe oder veränderte Rahmenbedingungen, sollten Pflegeberatende in der Lage sein, Inanspruchnehmende adäquat, individuell und angemessen beraten zu können. So verweisen Strube-Lahmann et al. [13] darauf, dass im Rahmen der COVID-19-Pandemie Pflegeberatende häufig erste Anlaufstelle für Fragen rund um das Thema COVID-19 waren. Folglich erscheint es notwendig, Pflegeberatende auf kurzfristig entstehende Beratungsbedarfe vorzubereiten und entsprechend ausreichende Informationen bereitzustellen, um vulnerable Menschen angemessen versorgen zu können.

## Limitationen

Ein Bezug zwischen Bedarfen und einzelnen Pflegegraden ist aufgrund der Umfrage nicht möglich. Allerdings wäre dies wünschenswert, um die individuellen Bedarfe noch genauer ermitteln zu können. Infolgedessen sollte dies Gegenstand nachfolgender Erhebungen und Analysen sein. Die Analyse der teilnehmenden Pflegeberatenden zeigt eine in Bezug auf die soziodemografischen Merkmale wie Geschlecht, Alter, Berufserfahrung etc. sehr heterogene Gruppe, Diese erscheint für die Grundgesamtheit der Pflegeberatenden in Deutschland nicht untypisch, auch wenn aufgrund der Online-Erhebung und der notwendigen datenschutzkonformen Anonymität der Teilnehmenden kein abschließendes Urteil über den Grad der Repräsentativität getätigt werden kann. Die Ergebnisse können daher – wenn überhaupt – nur auf den nationalen Raum übertragen werden, da die gesetzlichen Vorgaben und Leistungen der Pflegeberatung nach SGB XI § 7a nur in Deutschland Gültigkeit besitzen. Eine weitere Limitation dieser Studie besteht darin, dass ausschließlich die Sicht Pflegeberatender bezüglich der Beratungsbedarfe erfasst wurde, nicht jedoch die Perspektive der Personen, die die Pflegeberatung in Anspruch nehmen. In zukünftigen Studien könnten die Ergebnisse um diese weitere Perspektive ergänzt werden.

## Fazit für die Praxis

Um den Nutzen von Pflegeberatungen für die untersuchten Gruppen zu erhöhen, sollte die Priorisierung der Schwerpunktthemen der Pflegeempfangenden und Angehörigen sowohl bei dem Aufbau von Pflegeberatungsdatenbanken als auch bei den Beratungseinrichtungen vor Ort Berücksichtigung finden. Dabei müssen fortlaufend Studien zu spezifischen Bedarfen zur Pflegeberatung durchgeführt werden, um neue kurzfristig entstehende Bedarfe durch beispielsweise veränderte Rahmenbedingungen zu erfassen. Diese können dann unmittelbar in die Pflegeberatungsdatenbanken integriert werden, um eine angemessene, individuelle und bedarfsgerechte Beratung sicherzustellen.

## Supplementary Information




