# Supplementary material for: Support needs of care dependent individuals: Survey of general and specific needs for information, assistance and support of care dependent individuals and their relatives
Source: Z Gerontol Geriatr. 2022 May 4;56(4):301–8. [Article in German] doi: 10.1007/s00391-022-02068-w (PMC9066130; doi:10.1007/s00391-022-02068-w)
Supplement: Supplementary file 1 [file 391_2022_2068_MOESM1_ESM.docx]

**Supplement**

Teil 1

Zwei Pflegeberatende verfügten über eine Promotion. Weiterhin gaben 24,5 % an, über „sonstige“ Abschlüsse zu verfügen. Ein Großteil der Pflegeberatenden verfügte über Berufserfahrung in der praktischen Pflege. Von 268 Pflegeberatenden waren 118 Personen (43,1 %) länger als 10 Jahre und 34 (12,4 %) Personen 5 bis 10 Jahre in der praktischen Pflege tätig. 70 Pflegeberatende (25,5 %) konnten auf keine praktische Pflegeerfahrung zurückgreifen. 5,8 % bzw. 10,9 % der Befragten waren weniger als 1 Jahr bzw. 1 bis 4 Jahre in der praktischen Pflege tätig. Von sechs Personen lagen hierzu keine Angaben vor. In Hinblick auf die vorhandene Berufserfahrung als Pflegeberatende lagen Angaben von 267 Personen vor. Die Meisten verfügten über eine mehr als 10-jährige (33,6 %) bzw. 5- bis 10-jährige (27,7 %) Berufserfahrung in der Pflegeberatung.

Teil 2

Knoten 3: Bei 66 % der Pflegeempfangenden mit hohem oder sehr hohem Bedarf zur „Wohnraumberatung“ lag der MW des AIPE bei 8,3. Den höchsten Mittelwert bei den errechneten Endknoten hatten dabei diejenigen, die einen sehr hohen Bedarf im Hinblick auf „Mobilität“ hatten (Knoten 7; 9,2). Den insgesamt niedrigsten Wert von 5,7 trat bei Menschen auf, die sehr geringen bis mittleren Beratungsbedarf zu „Wohnraumberatung“ und „Ambulanten Leistungen“ hatten (Knoten 4). Den anteilig größten Endknoten von 29 % bildeten Personen mit sehr geringem bis mittlerem Beratungsbedarf zu „Wohnraumberatung“, aber hohem bis sehr hohem Beratungsbedarf zu „Ambulanten Leistungen“ (Knoten 5).

Teil 3

Knoten 3: Bei 95 % der Angehörigen/ Betreuenden mit mittlerem bis sehr hohem Bedarf zu diesem Thema lag der MW des AIAB bei 9,3. Gleichzeitig war dieser auch der höchste Mittelwert bei den errechneten sieben Endknoten. Der insgesamt niedrigste Wert von 6,9 fiel hingegen bei geringem oder sehr geringem Beratungsbedarf zum benannten Themenbereich (Knoten 2; 6,9) auf. Einen weiteren anteilig großen Endknoten von 24 % bildeten Personen mit mittlerem bis hohem Beratungsbedarf zu „Sozialrechtliche Aspekte, Ansprüche, Leistungen (SGB XI, Betreuungsrecht)“ bei zusätzlich hohem bis sehr hohem Beratungsbedarf zum Thema „Psychosoziale Situation“ und gleichzeitig geringem bis sehr geringem Beratungsbedarf zum Thema „Kulturelle und religiöse Aspekte“ (Knoten 27).
